# Evaluation of Reference Genes for RT-qPCR Expression Studies in Hop (*Humulus lupulus* L.) during Infection with Vascular Pathogen *Verticillium albo-atrum*

**DOI:** 10.1371/journal.pone.0068228

**Published:** 2013-07-12

**Authors:** Nataša Štajner, Sara Cregeen, Branka Javornik

**Affiliations:** Biotechnical Faculty, Agronomy Department, University of Ljubljana, Ljubljana, Slovenia; University of Texas Health Science Center, United States of America

## Abstract

Hop plant (*Humulus lupulus* L.), cultivated primarily for its use in the brewing industry, is faced with a variety of diseases, including severe vascular diseases, such as Verticillium wilt, against which no effective protection is available. The understanding of disease resistance with tools such as differentially expressed gene studies is an important objective of plant defense mechanisms. In this study, we evaluated twenty-three reference genes for RT-qPCR expression studies on hop under biotic stress conditions. The candidate genes were validated on susceptible and resistant hop cultivars sampled at three different time points after infection with *Verticillium albo-atrum*. The stability of expression and the number of genes required for accurate normalization were assessed by three different Excel-based approaches (geNorm v.3.5 software, NormFinder, and RefFinder). High consistency was found among them, identifying the same six best reference genes (*YLS8, DRH1, TIP41, CAC, POAC* and *SAND*) and five least stably expressed genes (*CYCL, UBQ11, POACT, GAPDH* and *NADH*). The candidate genes in different experimental subsets/conditions resulted in different rankings. A combination of the two best reference genes, *YLS8* and *DRH1*, was used for normalization of RT-qPCR data of the gene of interest (*PR-1*) implicated in biotic stress of hop. We outlined the differences between normalized and non-normalized values and the importance of RT-qPCR data normalization. The high correlation obtained among data standardized with different sets of reference genes confirms the suitability of the reference genes selected for normalization. Lower correlations between normalized and non-normalized data may reflect different quantity and/or quality of RNA samples used in RT-qPCR analyses.

## Introduction

Hop (*Humulus lupulus* L.) is cultivated primarily for its use in the brewing industry as a flavoring and stabilizing agent in beer. Hop cones, the female flower clusters, are covered with lupulin glands, rich in secondary metabolites, which give beer stability and bitterness (hop acids) and aroma (essential acids). Hop resin also contains polyphenols, which are gaining interest as pharmaceutical substances.

Hop growing, which is an important economic activity in some regions of Germany, USA, Czech Republic and Slovenia, is accompanied by plant protection against the most common hop diseases, such as powdery mildew (*Podosphaera macularis*), downy mildew (*Pseudoperonospora humuli*) and Verticilium wilt (*Verticillium albo atrum*). The first two diseases are successfully controlled by fungicides but Verticillum wilt is a challenge, since the most effective control is host resistance. Several wilt resistance hop breeding programs have been set up, particularly in Europe, where hop growers encounter severe Verticillium wilt outbreaks [1], [2]. In parallel with resistance breeding, molecular research into hop wilt resistance has been carried out, including differentially expressed gene studies.

Gene-expression analyses are increasingly important in many fields of biological research, including in the study of plant biotic stresses, since quantification of the level of gene expression gives an insight into patterns of plant defense during the various stages of stress. Measurement of the transcript abundance of genes implicated in plant defense with RT-qPCR enables the measurement of gene expression in many different samples for a number of genes of interest (GOI). Because of its high sensitivity in detecting differences in the abundance of mRNA, any source of variation, including sampling errors, template quality and amplification efficiency, biases the result. The normalization of the results of RT-qPCR by using internal reference genes is therefore essential for the accuracy of RT-qPCR analysis. The expression level of internal reference genes needs to remain constant under varying experimental conditions but there is evidence that transcript levels of housekeeping genes can vary considerably over developmental stages and under variable conditions [3], [4]. This variability in expression across experimental samples may be because these housekeeping genes not only participate in basic cell metabolism but also in other cellular processes [5], so reference genes need to undergo proper validation of their stability of expression. The same reference genes may not have stable expression for all genotypes and for all tissues and may depend on the applied treatment/conditions [3]; [6]; [7]. Accurate quantification of expression of the candidate genes requires prior screening and validation to select suitable reference genes for each genotype and tissue. However, many studies have used reference genes without proper validation of the stability of expression. Vandesompele et al. [4] reported that single reference normalization without previous validation and without using multiple housekeeping genes can provide error values of genes of interest up to 6.4-fold and can lead to misinterpretation, particularly in the case of small expression differences.

The expression stability and validation of reference genes suitable for normalization of qPCR data have been investigated in detail for various organisms, including plants such as rice [8]; [9] poplar [10], potato [11], soybean [12]; [13], *Arabidopsis* [6], coffee [14], sugarcane [15], tomato [16], grape [17] etc. In studies of gene expression in hop, the normalization strategy has mainly used only one housekeeping gene: the transcript levels of the O-methyltransferase gene were normalized by using *GAPDH* as the housekeeping gene [18], the expression levels of Valerophenone Synthase were normalized by the gene encoding the polyubiquitin protein [19] and the expression of *HlMyb3* transcription factor was normalized by the *7SL-RNA* reference gene [20]. In the most recent study of hop, 6 housekeeping genes were tested across different hop tissues of female genotypes and three of them (*GAPDH, DRH1, 7SI-RNA*) were used for normalization of expression of *Hua* enhancer 1 transcript factor [21].

Suitable reference genes for gene expression studies of stress conditions have not yet been defined for hop, so we selected a set of 23 reference genes commonly used for validation with different plant species [6]; [10]; [11]; [14]; [16]. The evaluation was carried out on hop plants inoculated with the soil-born vascular pathogen *Verticillium albo-atrum,* which causes considerable economic damage to hop production. Hypocotil tissues of susceptible and resistant hop cultivars at three different time points were used as experimental material. The procedure for accurate normalization of gene-expression data based on multiple reference genes is outlined. The most stable reference genes tested across different experimental stages were further used in the validation analysis of expression of the pathogenesis-related *PR-1* gene, which is one of the genes induced in the response of hop to infection. The normalized expression values of the *PR-1* gene were further compared to non-normalized expression levels in order to highlight the differences and the important role of data normalization.

## Materials and Methods

### 1.1 Plant Materials and Stress Treatments

The expression stability of 23 genes was tested on different hop samples; *V. albo-atrum* hop isolate (pathotype PV1) was used to inoculate 6-week old plants of susceptible hop cv. Celeia and resistant cv. Wye Target. Plants were inoculated by the root dip method [1] and were grown in a growth chamber. The stems of 6-week old plants were sampled within the first 10 cm above the soil; fresh samples were immediately frozen in liquid nitrogen and kept at −80°C until RNA isolation. Plants were sampled 10, 20 and 30 days post inoculation (dpi) to monitor fungus colonization. The samples were designated: 10C+, 20C+, 30C+ ( = infected cv. Celeia at 10, 20 and 30 dpi); 10W+, 20W+, 30W+ ( = infected cv. Wye Target at 10, 20 and 30 dpi). Control plants, which were inoculated with sterile distilled water and sampled at the same time points, were designated 10C−, 20C−, 30C− ( = non-infected cv. Celeia at 10, 20 and 30 dpi) and 10W−, 20W−, 30W− ( = non-infected cv. Wye Target at 10, 20 and 30). For confirmation of successful infection, a re-isolation test was carried out in addition to symptom assessment, all according to the protocol of [1].

### 1.2 Total RNA Extraction

RNA was extracted from infected and control plants by the TRIzol protocol (Life Technologies, Invitrogen). RNA concentrations were quantified by BioPhotometer, according to the manufacturer’s instruction (Eppendorf, Germany). The purity of the total RNA extracted was determined as the 260/280 nm ratio and the integrity was checked by electrophoresis in 1% agarose gel.

### 1.3 Primer Design

We selected twenty-one gene sequences that are described in the literature as the most common reference genes for plant species: *Arabidopsis thaliana* [6]; [10], *Solanum tuberosum* [11], *Lycopersicon esculentum* [16] and *Coffea* [14] (Table 1). Sequences were searched against locally formatted PlantGDB Assemblies for *Humulus lupulus* [22] and the BLASTN algorithm was used to find the best hit against sequences from other species. Twenty-one primer pairs were designed from these sequences (Table 1) (110 bp max length of the amplicon, optimal Tm at 60°C, GC% between 30% and 80%) with Primer Express 3.0.0 Applied Biosystems software. Primer sequences and the names of the genes are given in Table 1. We also used two published primers of the genes *7SLRNA* (7SL component of the signal recognition particle) and *DRH1* (DEAD box RNA helicase), which have been recently used as internal references in hop [21] (Table 1).

**Table 1:** Primer sequences of 23 reference genes that were designed for qPCR amplification.

Geneabbr.	Gene description	Homolog locus (Reference)	Accession number	BLASTXscore/E val.	Primer sequences (forward/reverse)
***ACT11***	Putative actin 11	*Arabidopsis thaliana* [10]	NM_112046	642/0.0	5′-GTGTCAGCCACACTGTTCCAA-3′ 5′-GTCACGACCTGCCAAGTCAA-3′
***TUB***	Tubulin alpha-5 chain	*Arabidopsis thaliana* [10]	NM_121983	517/e-146	5′-GAGACAAGTTCAGGCAAGCATGT-3′ 5′-GCGGTACTTGCCGGTTCTAA-3′
***UBQ11***	Ubiquitin 11	*Arabidopsis thaliana* [10]	NM_001125464	492/e-139	5′-GGTTCGGTCTCAGCTTTTGG-3′ 5′-TTATTTTCTGCAAAAGGCTTAACAAC-3′
***EF1B***	Elongation factor 1-beta	*Arabidopsis thaliana* [10]	AY065159	147/3e-035	5′-GGGTATCAGGCTTCCAAGGAT-3′ 5′-GTACCAACGAGACGCGTTCA-3′
***BTUB***	Beta-tubulin (βtub)	*Solanum tuberosum* [11]	Z33382	396/e-110	5′-CCCTGGTCAGCTCAATTCTGA-3′ 5′-GGTGCGAATCCAACCATGA-3′
***EF1A***	Elongation factor 1-alpha	*Solanum tuberosum* [11]	AB061263	1330/0.0	5′-CCCCAGGACATCGTGACTTT-3′ 5′-CTCCGGTGGTGGAGTCAATAA-3′
***POAB***	L2 cytoplasmic ribosom. protein	*Solanum tuberosum* [11]	CK259681	258/9e-069	5′-GTGACCGAGGTGTCCTTGCT-3′ 5′-GCACCAGATGGAAGCTTGACT-3′
***POAC***	Poact24	*Solanum tuberosum* [11]	CK270447	200/2e-051	5′-AAGACGATCGCATAGCGAGAA-3′ 5′-TCCAGAAGCAACGTAGTGATGTC-3′
***POACT***	Poac58 gene for actin	*Solanum tuberosum* [11]	X55749	369/e-101	5′-TCGGTTGAGAAGAGCTACGAGTT-3′ 5′-CGATCATGGATGGCTGGAA-3′
***CYCL***	Cyclophilin	*Solanum tuberosum* [11]	AF126551	289/2e-078	5′-CACCGGTCCCGGCATT-3′ 5′-CTTCCCATCGAGCCACTCA-3′
***NADH***	NADH dehydrogenase F	*Humulus lupulus* [21]	AY289251	307/3e-083	5′-AAGCTCCTTTCACTGCTTCTTACAG-3′ 5′-TCCAACTCTTCAGATGTCCTACCA-3′
***GAPDH***	Glyceraldehyde 3-phosphate dehydrogenase	*Coffea canephora* [16]	SGN-U347734^a^	638/0.0	5′-TGGAATGTCTTTCCGTGTTCCT-3′ 5′-GATGGCTTTTTTGATCTCCTCGTA-3′
***RPL7***	60S ribosomal protein L7	*Coffea canephora* [14]	SGN-U351477^a^	159/7e-039	5′-TTCATTATTCGCATCCGTGGTA-3′ 5′-TTAAGGAACACACCATTGAATATCTGT-3′
***CYS***	Cysteine proteinase	*Coffea canephora* [14]	SGN-U352616^a^	115/2e-025	5′-CTGAGCAAGAGTTGGTGGATTG-3′ 5′-ATGCCGCCATTGTTGATGAT-3′
***TBP***	TATA binding protein	*Solanum lycopersic.* [16]	AK329831	358/4e-099	5′-AACCAGACTCAACGGTCGTCTT-3′ 5′-CGATTGACAACTCACCGTGAA-3′
***TIP41***	TIP41-like family protein	*Solanum lycopersic.* [16]	BT014035	186 4e-047	5′-GGAGGTGGAGGTTGACGATAAC-3′ 5′-GGACTCGACCTCCCAACCAT-3′
***SAND***	SAND family protein	*Arabidopsis thaliana* [6]	NM_128399	82/2e-015	5′-ACGAGCTGCTGTCGTTTGG-3′ 5′-ACTACTTCCTCTTTCACCAGCATACC-3′
***CAC***	Clathrin adaptor complexes medium subun.	*Solanum lycopersic.* [16]	SGN-U314153^b^	100/8e-021	5′-CTGGCCATGTGAGAATTTCCTT-3′ 5′-CTCGCAATTTTTGTCACTACAACA-3′
***ACCG8***	Acetyl-coA carboxylase	*Brassica napus* [40]	X77576	92/2e-017	5′-GCAGCTTTTGCCTTTATACACTCA-3′ 5′-GTCCACTCCAGAACTTCTTTAACAACT-3′
***YLS8***	Yellow leaf specific protein 8	*Arabidopsis thaliana* [6]	NM_120912	323/2e-088	5′-CGTACCTGCTTCCACATTTGC-3′ 5′-TCCCAGTCGTGGCCAAAA-3′
***AT1G1***	Phosphatase 2A regulat. subun. *(PP2AA3)*	*Arabidopsis thaliana* [6]	NM_001198052	507/e-143	5′-AGCAAATCAATTGTACGAGCTTTGT-3′ 5′-TGCCTCGTTATCTGGAAGCA-3′
***7SLRNA***	7SL component of the signal recognition particle	*Humulus lupulus * [*21*]	-	-	5′-TGTAACCCAAGTGGGGG-3′ 5′-GCACCGGCCCGTTATCC-3′
***DRH1***	DEAD box RNA helicase	*Humulus lupulus * [*21*]	-	-	5′-CCAACCTACTGGGCTTCGAC-3′ 5′-CAGAATGGGTATGATCGGGC-3′

a Unigene accession number according to the SOL Genomics Network (Solanaceae Genome Project - SOL) [http://www.sgn.cornell.edu/content/coffee.pl].

Differential protein analysis after infection of hop plants with *Verticillium albo-atrum* revealed an upregulated protein, which was identified as PR-1 (pathogenesis-related protein 1). The highest ranked hit was the sequence from *Eutrema wasabi* (GenBank accession BAF03262). On the basis of this sequence, we identified the hop PR-1 gene from the hop EST database (http://www.plantgdb.org/download/download.php?dir=/Sequence/ESTcontig/Humulus_lupulus) with tblastn search. The primers based on the hop sequence were developed using the program Primer Express 3.0.0 Applied Biosystems software and were as follows: forward (5′-GAAGGTACCCTTATTGTTGTTGCA-3′), reverse (5′-GTTTGCGGGCACTACACTCA-3′).

### 1.4 Two-step Real-time RT-PCR

In order to synthesize single stranded cDNA from total RNA, one microgram of each bulk RNA sample (generated from 5 individual plants per experiment point) was reverse transcribed to cDNA with a High Capacity cDNA Reverse Transcription Kit (Applied Biosystems, Foster City, USA) using random hexamers. Real-time PCR was performed using Fast SYBR Green Technology in the ABI PRISM 7500 Fast Sequence Detection System (Applied Biosystems, Foster City, USA). A master mix for each PCR run was prepared with SYBR Green PCR Core Reagents (Applied Biosystems, Foster City, USA). Final concentrations, in a total volume of 20 µl, were: 10 µl fast SYBR Green Master Mix, 10 ng of cDNA and 300 nM of each specific sense and anti-sense primer. The following amplification program was used: 95°C 20 s, 40 cycles at 95°C for 3 s followed by 60°C for 30 s. All samples were amplified in triplicate from the same RNA preparation and the mean value was calculated from three values. The amplification efficiency determined for each housekeeping gene was calculated using the slope of the regression line in the standard curve. For this, cDNA samples were bulked and serial dilutions from bulked samples were then used as the PCR template in a range of 50, 12.5, 3.12, 0.8, 0.2 and 0.05 ng. The corresponding real-time PCR efficiencies were calculated with ABI 7500 software (Version 2.0.4).

Amplified products (Table S1) were verified on a 2% agarose gel for their correct sizes and melting curve experiments showed whether a single amplification had been obtained for each gene. The RNA amplification levels for different reference genes and for all hop samples were determined as Ct (cycle threshold) values, which are the number of cycles needed for the amplification to reach a threshold fixed in the exponential phase of the PCR reaction [23]. The Ct values were calculated for each time-point to investigate the expression levels of each reference gene.

### 1.5 Statistical Analysis of Gene Expression Stability

The suitability of candidate control genes was evaluated by applying three different statistical approaches for the evaluation of expression data. In the first approach, using geNorm v.3.5 software [4] (http://medgen.ugent.be/~jvdesomp/genorm/), Ct values were transformed into relative quantities by the comparative Ct method (2^∧^(ΔCt)), whereby the sample with the lowest Ct value was used as calibrator. These reference gene quantities were then used as input data for geNorm to calculate the expression stability value (*M*) for each gene on the basis of the average pairwise variation of a particular gene compared to all other control genes in the set of samples. The geNorm program also enables calculation of the minimum number of control genes needed for accurate normalization. The procedure is based on the determination of a normalization factor (NF), expressed as a geometric mean of the reference gene quantities. The NF for each sample is first calculated with the two top ranked genes and the most stable remaining gene is then stepwise included, with the NF being recalculated at each step. The effect of including an additional gene is the pair-wise variation of two sequential NFs (Vn/n+1) estimated as the standard deviation of the logarithmically transformed NFn/NFn+1 ratios. A cut-off value for pairwise variation (0.15) is determined and below this value no additional gene is required for data normalization [4].

In the second evaluation approach, in which NormFinder software [24] (Microsoft Excel Add-in, available on the internet: http://www.mdl.dk/publicationsnormfinder.htm) was used, Ct values were log-transformed and imported into the application. This strategy is based on a mixed-linear effect model that enables estimation of the intra- and intergroup variation, which is then combined into a stability value. Candidate control genes with the minimum intra- and intergroup variation have the lowest stability values and are top ranked. For this purpose, our experimental data were subdivided into two coherent groups, firstly on the basis of different genotypes (cv. Celeia and cv. Wye Target) and secondly on the basis of experimental conditions (infected vs. control plants), with each subgroup consisting of 6 samples. The results of this algorithm rank the candidate normalization genes according to their expression stability in a given sample set and given experimental design.

The third evaluation approach was performed with the application RefFinder (http://www.leonxie.com/referencegene.php), which is a user-friendly web-based comprehensive tool developed for evaluating and screening reference genes of different experimental datasets. It integrates the currently available major computational programs (geNorm [4], Normfinder [24], BestKeeper [25] and the comparative ΔCt method [26]) to compare and rank the tested candidate reference genes.

The statistical analysis (ANOVA for four factor experiment) and the expression heat map were performed with program R (http://www.R-project.org/).

## Results

### 2.1 RT-qPCR Analysis

The sensitivity of SYBR Green-based RT-qPCR requires the use of the lowest concentrations of primers and target (cDNA) possible, in order to minimize the formation of non-specific amplification products. The primer and cDNA concentrations that yielded the highest target product (the lowest Ct values) without compromising the efficiency were therefore identified and the optimal primer concentration for all candidate genes proved to be 200 nM, and cDNA concentration 0.5 ng/µl. Verification of the amplified product of the tested genes was performed by agarose gel electrophoresis, which confirmed the expected sizes of amplicons. Melting curve analysis confirmed the amplification of a single product (data available on request). A six-fold dilution series of the bulked samples was used to prepare standard curves, from which primer efficiency was calculated using the formula E = 10^−1/SLOPE^. The PCR efficiencies for all amplified gene regions were between 92% and 110%, thus showing that the PCR reaction was not inhibited.

### 2.2 Expression Levels of Reference Genes

**Figure 1:**
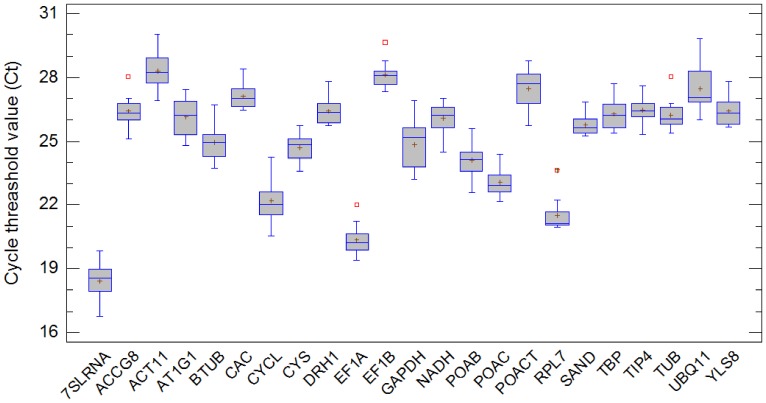
Expression data displayed as Ct values for each reference gene in all 12 samples. The figure summarizes data through 5 statistics: the markers in the box indicate average cycle threshold (Ct) values, squares indicate the presence of outliers, the box corresponding to an individual reference gene indicates the 25% and 75% percentiles, whiskers represent the maximum and minimum values and the median is depicted by the line across the box.

An overview of the relative abundance and expression levels of the candidate reference genes is given in Figure 1, with box-and-whisker plots that summarize data (n = 12) for each reference gene; each box corresponding to an individual reference gene indicates the 25% and 75% percentiles, whiskers represent the maximum and minimum values and the median is depicted by the line across the box. Markers indicate average cycle threshold (Ct) values. The outliers marked with a red square all correspond to the sample 30W− ( = non-infected cv. Wye Target at 30 dpi). The 23 genes show a wide range of Ct values, ranging in average from 18.4 for 7SLRNA to 28.3 for *ACT11*, which shows that *7SLRNA* transcript is approximately 1000–fold more abundant than *ACT11* in the hop transcriptome.

**Figure 2:**
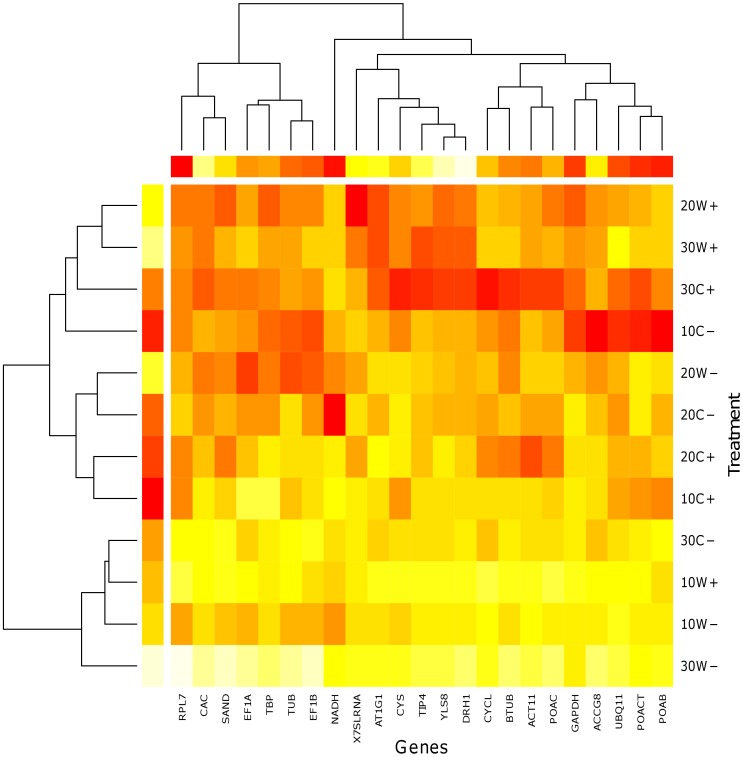
Expression heat map illustrating the relative expression levels and clustering for two genotypes (W, Wye Target and C, Celeia) according to the treatment (+, infection −, control). Each colored cell in the heat map represents the standardized relative gene expression value for a treatment and sample. The largest gene expression values are displayed in red color, intermediate values in shades of yellow and orange and the smallest values in light yellow.

Expression levels were measured at three different experimental time points/days post inoculation with *V. albo atrum* to allow full development of disease syndromes accompanied by the transcriptome changes. Two different genotypes, susceptible cv. Celeia and resistant cv. Wye Target, were chosen for analysis to capture the different responses to the pathogen that might influence the stability of reference gene expression. An approximately 10000-fold expression difference appeared between the most and least abundantly expressed genes (for individual samples). The gene-specific variation of expression levels resulted in the highest difference (14-fold) for *UBQ11* and the lowest (3-fold) for *SAND*. We also determined sample-specific relative expression levels for cv. Celeia and cv. Wye Target, ranked from the lowest to the highest as follows 30W−, 10W+, 30C−, 10W−, 10C+, 20C+, 20C−, 20W−, 30W+, 20W+, 10C−, 30C+ with a 5-fold maximum difference observed between samples 30C+ and 30W−, for which the average expressions were 0.17 and 0.82, respectively. Relative expression values were normalized according to the highest expressed sample for a single gene and are presented with heat map using terrain scale pallete inside program R. The largest gene expression values are displayed in red color, intermediate values in shades of yellow and orange and the smallest values in light yellow (Figure 2).

### 2.3 Gene Expression Stability Analysis

The gene expression stability of candidate reference genes was evaluated by using different approaches. Analyses using the RefFinder integration tool demonstrated an overall comprehensive ranking of reference genes integrated from three different algorithms. The reference genes calculated on the basis of different algorithms are presented in Table 2 and are ranked from the most stable to the least stable gene. Based on the rankings from each algorithm and its stability values, the geometric mean of the weights of individual genes was calculated for an overall final ranking. According to this ranking, *DRH1* gene was the most stable expressed gene in our set of samples, followed by the next five genes, which are identical by all other algorithms, but with slightly different rankings (Table 2).

**Table 2:** Integrated table of reference gene expression stability values based on different algorithms.

GeNorm		NormFinder		Delta Ct		Best Keeper		Comprehensive ranking^a^	
*Gene name*	*Stabil. value*	*Gene name*	*Stabil. value*	*Gene name*	*Aver. of STDEV*	*Gene name*	*Stabil. value*	*Gene name*	*Geomean*
*DRH1*	0.12	*DRH1*	0.198	*DRH1*	0.49	*SAND*	0.36	*DRH1*	1.41
*YLS8*	0.12	*CAC*	0.204	*CAC*	0.49	*CAC*	0.45	*CAC*	2.38
*TIP41*	0.15	*POAC*	0.211	*POAC*	0.5	*EF1B*	0.47	*SAND*	3.5
*CAC*	0.21	*YLS8*	0.261	*YLS8*	0.52	*DRH1*	0.48	*YLS8*	3.56
*POAC*	0.251	*SAND*	0.286	*SAND*	0.53	*POAC*	0.48	*POAC*	3.87
*SAND*	0.278	*TIP41*	0.298	*TIP41*	0.53	*TIP41*	0.49	*TIP41*	5.05
*TBP*	0.33	*BTUB*	0.318	*BTUB*	0.56	*EF1A*	0.52	*EF1B*	6.45
*EF1B*	0.359	*ACCG8*	0.353	*EF1B*	0.57	*ACCG8*	0.53	*BTUB*	9.48
*EF1A*	0.379	*EF1B*	0.362	*TUB*	0.58	*TUB*	0.54	*ACCG8*	9.55
*TUB*	0.394	*RPL7*	0.367	*ACCG8*	0.58	*YLS8*	0.54	*TUB*	9.72
*BTUB*	0.407	*TUB*	0.373	*RPL7*	0.58	*CYS*	0.54	*EF1A*	10.16
*RPL7*	0.417	*TBP*	0.386	*TBP*	0.59	*POAB*	0.55	*TBP*	11.27
*ACCG8*	0.427	*EF1A*	0.413	*EF1A*	0.6	*RPL7*	0.55	*RPL7*	11.45
*CYS*	0.443	*CYS*	0.423	*CYS*	0.62	*NADH*	0.56	*CYS*	13.18
*7SLRNA*	0.463	*ACT11*	0.539	*ACT11*	0.7	*BTUB*	0.61	*POAB*	15.81
*ACT11*	0.487	*7SLRNA*	0.55	*7SLRNA*	0.7	*TBP*	0.64	*7SLRNA*	15.98
*AT1G1*	0.508	*POAB*	0.553	*POAB*	0.71	*7SLRNA*	0.65	*ACT11*	16.38
*POAB*	0.528	*AT1G1*	0.586	*AT1G1*	0.73	*AT1G1*	0.76	*AT1G1*	17.74
*CYCL*	0.549	*CYCL*	0.611	*CYCL*	0.75	*CYCL*	0.77	*CYCL*	19
*UBQ11*	0.571	*UBQ11*	0.645	*UBQ11*	0.78	*ACT11*	0.77	*NADH*	20.32
*POACT*	0.591	*POACT*	0.678	*POACT*	0.81	*POACT*	0.86	*UBQ11*	20.48
*GAPDH*	0.614	*GAPDH*	0.748	*GAPDH*	0.87	*UBQ11*	0.89	*POACT*	21
*NADH*	0.639	*NADH*	0.804	*NADH*	0.9	*GAPDH*	0.97	*GAPDH*	22.25

a Comprehensive ranking based on geometrical mean of four algorithms (GeNorm, NormFinder, Delta Ct and Best Keeper) performed with RefFinder approach.

**Figure 3:**
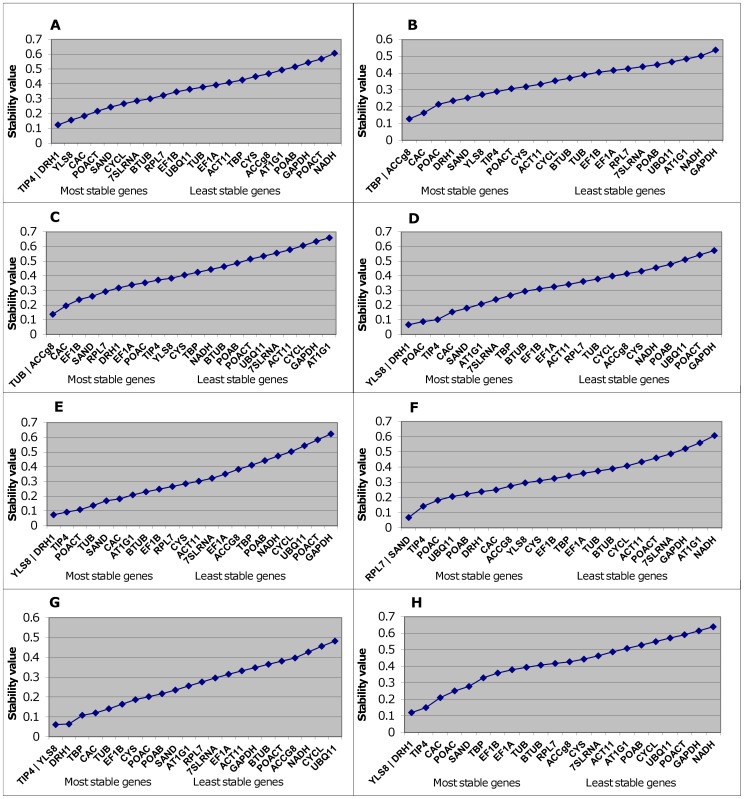
Average expression stability values (M) calculated by geNorm. The genes are ranked according to their expression stability; the least stable genes are on the left, and the two most stable genes are on the right. The samples are divided into sub-groups: (A) susceptible plants of cv. Celeia; (B) resistant plants of cv. Wye Target; (C) plants infected with *Verticillium albo-atrum*; (D) control (non-infected) plants; (E) plants evaluated 10 days post inoculation with fungi; (F) plants evaluated 20 days post inoculation with fungi (G) plants evaluated 30 days post inoculation with fungi (H) all plant samples.

On the basis of the geNorm calculations, the stability values (M) were analyzed over the whole set of samples and also across seven subsets (Figure 3). The systematic validation of candidate genes demonstrated that none of them performed consistently well for all experimental conditions and that the stability of the genes varied according to the conditions (Figure 3). Nevertheless, the combination *DRH1*/*YLS8* occurred as the most stable in two different subsets, in non-infected plants (controls) and in plants evaluated at 10 days post inoculation. In plants evaluated 30 days post inoculation, *DRH1* also has nearly the same stability value as *YLS8*/*TIP4* and can also be considered to be one of the most stable reference genes. The gene pair *YLS8*/*DRH1* is the only one that occurred more than once in different subsets and also proved to be the most stable over the whole set of samples (Figure 3h).

**Figure 4:**
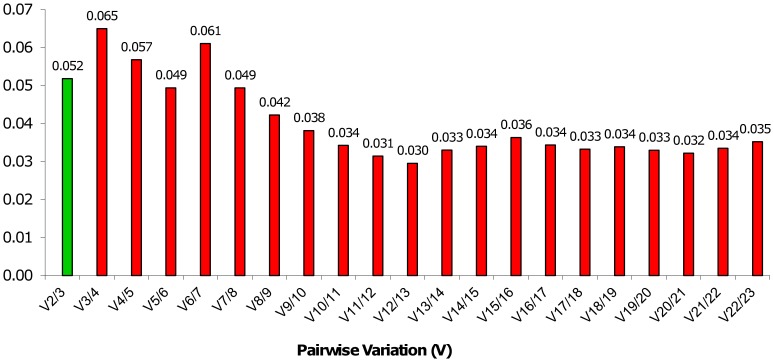
Pairwise variation (V) between the normalization factors (NFn and NFn+1) to determine the optimal number of reference genes. V2/3 represents the pairwise variation between the NF values estimated for the two genes YLS8 and DRH1 and the NF values estimated for the three genes, YLS8, DRH1 and TIP41, and adding all 23 genes in the same way.

The optimal number of genes that are necessary for accurate normalization was determined for the whole set of samples (Figure 4). The pair-wise variation of two sequential normalization factors (Vn/n+1) shows that two reference genes are sufficient for the calculation of the normalization factor, since the V2/3 value is 0.052, which is below the cut off value of 0.15 [4]. The low (Vn/n+1) value showed that the inclusion of the (n +1)^th^ gene had no significant effect.

**Figure 5:**
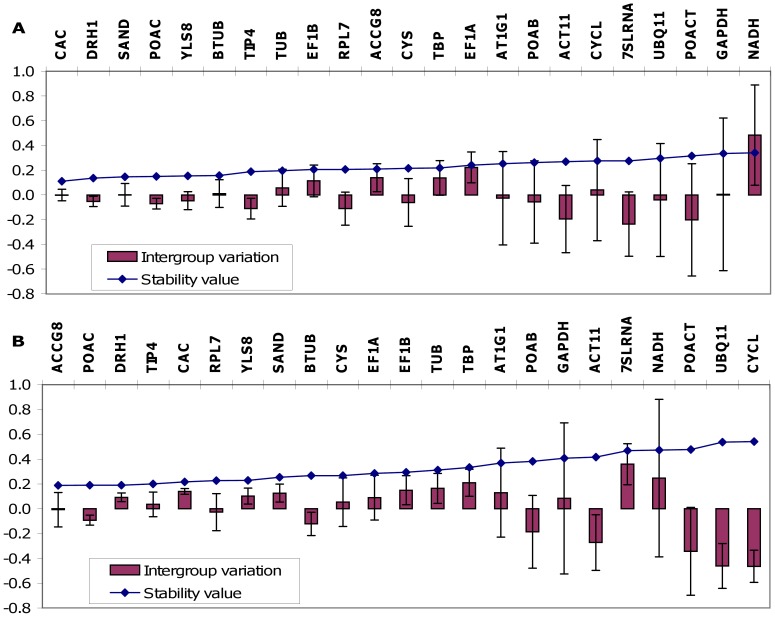
Stability values and estimated inter- and intra-group variances for different grouping calculated by NormFinder. (A) infected vs. non-infected plants and (B) susceptible vs. resistant plants. The intra-group variation is represented as error bars.

NormFinder analysis indicated *DRH1* to be the most stable gene over the whole set of samples (Figure S1). Taking into consideration the grouping of the samples, the best single genes were *CAC* and *ACCg8* for infected vs. non-infected and susceptible vs. resistant genotypes, respectively (Figure 5). The best combination of two most stable reference genes in terms of intra– and inter–group variation proved to be *SAND*/*CAC* when the samples were grouped as infected vs. non-infected (5A) and *POAC*/*DRH1* when the samples were grouped as susceptible vs. resistant (Figure 5B). The optimal number of reference genes based on accumulated standard deviation calculated by NormFinder comprises 14 reference genes (Figure S2).

### 2.4 Validation of Reference Genes

**Figure 6:**
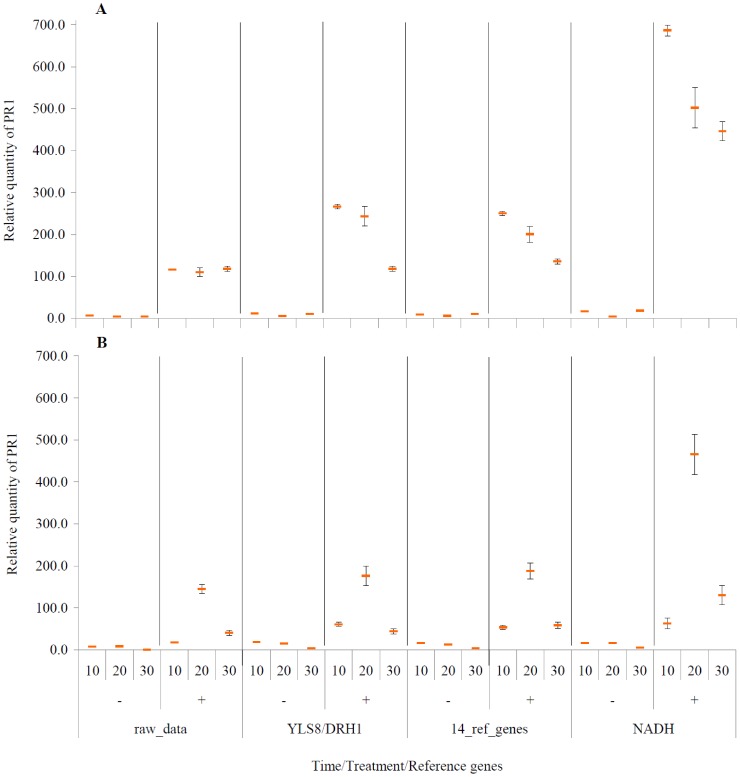
Expression analysis of PR-1 gene in response to *V. albo atrum* infection. (A) cv. Celeia and (B) cv. Wye Target for infected (+) and control plants (−) at three different experimental time points (10, 20, 30 dpi). Expression values were normalized with three normalization factors: YLS8/DRH1 (two best reference genes), 14 best reference genes (resulting from NormFinder) and least stable gene (*NADH*) and are displayed as normalized and non-normalized (raw) values. Bars indicate standard errors.

In order to validate the selected genes for normalization of expression data for biotic stress conditions in hop, the expression level of pathogenesis related gene *PR-1* was measured in resistant and susceptible cultivars challenged by *V. albo-atrum*. The effect of biotic stress is illustrated for raw and normalized relative quantification values (Figure 6). The expression of PR-1 was normalized by applying either the two (*YLS8*/*DRH1*) or 14 best reference genes, identified by GeNorm and NormFinder, separately. *NADH,* as the least stable gene according to both, GeNorm and NormFinder, was included in the analyses to verify its influence on the resulting normalization.

The non-infected (control) plants of cultivars cv. Celeia (C−) and cv. Wye Target (W−), expressed very low values for *PR-1* expression at all three dpi time points (Figure 6). The expression of *PR-1* for infected cv. Celeia (+, Figure 6A) increased significantly at 10 dpi (23 times compared to non-infected plants) and gradually declined over 20 and 30 dpi. The expression of PR-1 for infected cv. Wye Target (W+, Figure 6B) significantly increased at 20 dpi and then declined at 30 dpi to a level similar to that for 10 dpi. The difference of transcript abundance of infected cv. Celeia between 20 and 30 dpi was 2 times higher when normalized against the two most suitable reference genes, compared to raw values. The level of expression for raw data was nearly the same within all three time points, which means that the expression at 10 and 20 dpi is underestimated compared to normalized data. The expression levels of infected cv. Wye Target were more similar between normalized (2 and 14 best references) and raw data compared to cv. Celeia, but again, raw expression value at 10 dpi was underestimated compared to normalized values. Normalization of *PR-1* data with *NADH* chosen as the least appropriate reference gene resulted in significantly higher expression and significantly different expression ratios than with values normalized with 2 geNorm and 14 NormFinder selected genes. This demonstrates how an improper reference gene used for data normalization can affect the values and lead to misinterpretation of the results.

The correlation coefficients of expression levels between 1) *YLS8/DRH1*-normalized values and 14-gene-normalized values, 2) non-normalized and 14-gene-normalized values and 3) non-normalized and *YLS8/DRH1*-normalized values were 0.99, 0.94 and 0.90, respectively. The correlation coefficients between *NADH*- and *YLS8/DRH1*-normalized values and *NADH*- and 14-gene-normalized values were 0.67 and 0.74, respectively and confirm the above findings regarding the inapplicability of this gene for normalization of data.

The statistical analysis of data was performed with four factor ANOVA for fixed factors: cultivar (Celeia and Wye Target), days post inoculation (3 DPI time), infection (control and infected plants) and standardization (raw data, *YLS8/DRH1* genes, 14 ref.genes, *NADH* gene). Logarithmic transformation was used to achieve the ANOVA assumption about equality of variances. ANOVA showed that all interactions in four factor experiment are statistically significant (Table S2), so Tukey’s multiple comparisons test was used to test the differences between averages of 48 treatments (Table S3). There is no statistically significant difference in relative *PR1* levels when using *YLS8/DRH1* vs. 14 ref.genes, which was expected and confirms the validation and proper selection of the two best reference genes. The differences are statistically significant between raw data and data normalized with *YLS8/DRH1* genes for infected Celeia plants at 30 dpi and for infected Wye Target plants at 20 and 30 dpi. When using *NADH* compared to *YLS8/DRH1* standardization genes, the differences are statistically significant for all Celeia samples and for Wye Target samples at 20 and 30 dpi.

## Discussion

### 3.1 Expression Levels of Reference Genes

The gene-specific variation of expression levels based on the relative quantities of analysed samples within a specific gene differed somewhat from the results of expression stability analyses; Genes *SAND* and *UBQ11* showed the lowest and the highest expression variation within the given set of samples, respectively but, based on the different algorithm calculations, they were not ranked as the most and least stable genes, which highlights the importance of algorithm based calculations. Sample specific relative expression levels revealed no time or genotype or biotic stress dependent expression variability and it can be assumed that the obtained variability depends only on DNA quality and quantity.

### 3.2 Gene Expression Stability Analysis

The systematic validation of candidate genes based on the susceptibility of the genotype, biotic stress and time post inoculation with Verticillium wilt resulted in a different ranking of genes based on stability values (Figure 3). The different ranking of candidate genes in different experimental subsets emphasizes the need for detailed reference gene analysis for different experimental conditions, which is in accordance with previous studies reporting inter-tissue and experiment-dependent variation in gene stability [3]; [27]; [28] and not just variation between specific plant species. Systematic validation and the use of at least two validated reference genes involved in distinct cellular functions has been proposed by different studies [4]; [5], since no single gene can act as a universal reference. Based on the accumulated standard deviation (Acc. SD), NormFinder recommended the 14 most stable reference genes for accurate data normalization in a given set of samples (Figure S2). Similarly, within GeNorm, the lowest pair-wise variation of normalization factors (V12/13) is obtained when the 12 most stable genes are included in the analysis but since the stability values for the tested genes and the pair-wise variations are very low and are all under the cut-off value (0.15), the two most stable genes are considered to be sufficient for reliable normalization of hop samples under biotic stress.

Different approaches resulted in slightly different rankings of gene stability but high consistency was found among them.The NormFinder algorithm, which is based on standard deviation and, in contrast to GeNorm, does not consider the co-regulation of all other genes, revealed the same best gene as GeNorm; *DRH1* was found to be the most stable. In contrast to GeNorm, NormFinder can also take into consideration the groupings of samples, such as, in our experiment, infected and non-infected plants, or two different genotypes (susceptible and resistant). NormFinder calculates both intragroup variance, describing the stability of gene expression within each group, and intergroup variance, which describes the stability of gene expression between groups. The most stable genes with low stability values displayed small error bars and inter-group variances close to zero (Figure 5), which is evidence of comparable stability in both subsets of the experiments. Based on the two different grouping conditions, the single genes *CAC* and *ACCg8* were revealed to be optimal, or *SAND*/*CAC* and *POAC*/*DRH1* in the case of a combination of the two best reference genes. The differences obtained as the consequence of the grouping influence are well shown in Figure 5. In the second grouping (infected vs. non-infected plants), the optimum pair of genes (*POAC/DRH1*) does not include the optimum single gene (*ACCg8*), which is the consequence of the compensating expression of the resulting most stable pair, so that one gene is slightly over-expressed in one group but the other gene is correspondingly under-expressed in the same group and it therefore differs from the resulting best single gene [24]. The different genes/pairs obtained for a particular grouping show that the expression stability of reference genes depends on the experimental parameters, which should also be taken into consideration.

The majority of gene expression studies on hop have been performed using only one housekeeping gene [18]; [19]; [20]; only in the most recent study were 6 housekeeping genes tested across different hop tissues of female genotypes [21]. Comparing our study to the study of Maloukh et al. [21], the stability values of four out of six reference genes (*NADH*, *7SLRNA*, *DRH1*, *GAPDH*) were significantly lower in our experiment; for example, *DRH1* had a 7.5 times lower stability value in our experiment, although both experiments indicated that it is one of the most stable reference genes. The expression stability differences might be a result of different tissues analyzed in the compared experiments. The second observed difference relates to the ranking of the stability of the reference genes, e.g., *GAPDH* was found to be one of the two most stable genes in a previous study [21] but to be one of the least stable genes in our study. The regulation of reference genes is not only variety/cultivar specific but may also be tissue specific and influenced by the experimental conditions [29]; [30]; [11]. According to Vandesompele et al. [4], some genes have a relatively constant expression level among different tissues but others do not. This is the main reason for validating housekeeping genes for a specific genotype and experiment.

### 3.3 Validation of Reference Genes

The resulting expression pattern indicates that *PR-1* gene is involved in biotic stress after infection of plants with *V. albo-atrum* and is one of the genes representing the regulation of plant defense (Figure 6). The expression of PR-1 was significantly different between infected and non-infected plants and also between the two cultivars (susceptible and resistant), demonstrating a specific response of the genotype to biotic stress. Susceptible cv. Celeia exhibited the highest expression level of PR-1 at 10 dpi, 4.4-times higher than the expression level in resistant cv. Wye Target, which responded to stress only at 20 dpi. Higher induction of defense genes in susceptible than in resistant plants on infection with vascular wilt diseases such as Verticillium wilt has been previously reported [31].

Very similar relative transcript abundances (expression levels) were obtained for data normalized using the 2 or 14 best defined reference genes, in both genotypes, but expression levels were different if they were not normalized or if they were normalized with *NADH* (the least stable gene). The high correlation obtained among standardized data confirms the validity of the two reference genes (*DRH1*and *YLS8*) selected for normalization, but lower correlations between normalized and non-normalized data reflect differences in gene expression, which may derive from a different quantity and/or quality of RNA samples used in RT-qPCR analyses.

A few genes that were shown to be stably expressed in our study could be used as references for further studies on gene expression in hop. Different statistical approaches resulted in a slightly different ranking of these genes. We decided to use GeNorm for the reference algorithm, as the most widely used algorithm in recent published studies, and two genes, *YLS8* and *DRH1*, were therefore chosen for the normalization of GOI and for further studies.

*YLS8* (yellow leaf specific protein 8) encodes for the mitosis protein *YLS8* but the actual function of this gene is not known. It has been identified as a novel suitable reference in *A. thaliana* [7] and was also shown to be one of the most stable genes used for normalization in studies of *Havea brasilensis* in response to tapping, hormone and salicylic acid treatment [3]. This gene was shown to be highly stable in *Arabidopsis* exposed to increased metal concentrations [7] and constant in different tissues of cultivated peanut [32]. The same gene was found to be less stable in various cucumber organs exposed to heavy metal stress [33]. The *YLS8* gene was also excluded from the list of candidate reference genes for normalization of transcripts from virus-infected *Arabidopsis thaliana* [34], due to an increase in transcript accumulation in response to cabbage leaf curl virus (CaLCuV) of virus-infected *Arabidopsis* plants [35].

The *DRH1* gene (DEAD box RNA helicase) was previously used as a housekeeping gene in plant studies by de Boer et al. [36] but without any comparison to other housekeeping genes. In a previous study on hop [21], *DRH1* in combination with *GAPDH* were shown to be the most stable reference genes and therefore used in quantitative analysis of transcript accumulation of *Hua Enhancer1* transcription factor. The *DRH1* gene belongs to the RNA helicase superfamily II (SFII) [37] and, in addition to their housekeeping function in RNA unwinding, initiation of translation, RNA processing and ribosome assembly, some members of the RH family have been implicated in more specific processes during development and cell differentiation [38]. However, most of the RNA helicase evaluated in *A. thaliana* were shown to be housekeeping genes, since they were transcribed in all organs analyzed, with an overall similar transcript profile, except for two that exhibited plant-specific differences associated with photosynthetically active and photosynthetically inactive organs [38]. In contrast, functional studies published by Kant et al. [39] identified two genes encoding *Arabidopsis thaliana* DEAD-box RNA helicases as being down-regulated by multiple abiotic stresses (increased tolerance to salt, osmotic and/or heat stresses).

In conclusion, using three approaches (geNorm, NormFinder and RefFinder), the variation of the expression of 23 reference genes in hop under biotic stress conditions identified the same six most and five least stable reference genes, with slightly different rankings. The two genes, *YLS8* and *DRH1*, most stably expressed within the GeNorm ranking, were shown to be sufficient for accurate standardization of data on the basis of a low pair-wise variation between gene normalization factors and validation of the genes by *PR-1* expression gene analysis. The advantage of the NormFinder algorithm compared to the others is the possibility of estimating not only the overall expression variation of the candidate normalization genes but also the variation between and within sample sub-groups that were shown to be different. On the basis of gene expression analysis of *PR-1*, which is implicated in biotic stress, we outlined differences between normalized and non-normalized expression data and showed that suitable references are needed to eliminate the expression differences arising from different qualities and quantities of RNA.

Production of high quality hops requires controlling diseases and pests that could reduce the quality of yield or cause total crop loss, depending on the severity of disease. Verticillium wilt is a potentially damaging disease of hop and numerous other crops, including alfalfa, cherry, maple, mint, potato, as well as several herbaceous plants. The gene expression studies and reference gene validation of hop infected with Verticillium wilt applied in our research can considerably facilitate experimental analyses in plant defense and protection.

## Supporting Information

Figure S1 **Standard deviations (SD) for stability ranking of reference genes calculated by NormFinder.** One bar per gene represents the gene variability. The best reference gene is highlighted in red.
(TIFF)

Figure S2 **Determination of the optimal number of reference genes based on Acc. SD calculated by NormFinder.** The Acc. SD is plotted against number of reference genes in a line plot where the optimal number of reference genes is indicated in red.
(TIFF)

Table S1 **Amplicon sequences of 21 reference genes which were amplified in qPCR.**
(DOC)

Table S2 **The statistical analysis of data preformed with four factor ANOVA for fixed factors: cultivar (Celeia and Wye target), DPI (days post inoculation), infection (control and infected plants) and standardization (raw data, YLS8/DRH1 genes, 14 ref.genes, NADH gene).** F and Pr values indicate that all interactions in four factor experiment are statistically significant.
(DOC)

Table S3 **The average expression stability values of cultivars Celeia and Wye Target for infected (+) and control plants (−) at three different experimental time points (10, 20, 30 dpi).** Expression values were normalized with three normalization factors obtained from two best reference genes (YLS8/DRH1), 14 best reference genes and least stable gene (*NADH*) and were compared to non-normalized (raw) values. s_e_ represent standard errors. Different letters indicate statistical significance based on Tukey’s multiple comparisons test of individual averages of expression levels.
(DOC)
