# Supplementary figures and images for: Evaluation of Reference Genes for RT-qPCR Expression Studies in Hop (Humulus lupulus L.) during Infection with Vascular Pathogen Verticillium albo-atrum
Source: PLoS One. 2013 Jul 12;8(7):e68228. doi: 10.1371/journal.pone.0068228 (PMC3709999; doi:10.1371/journal.pone.0068228)

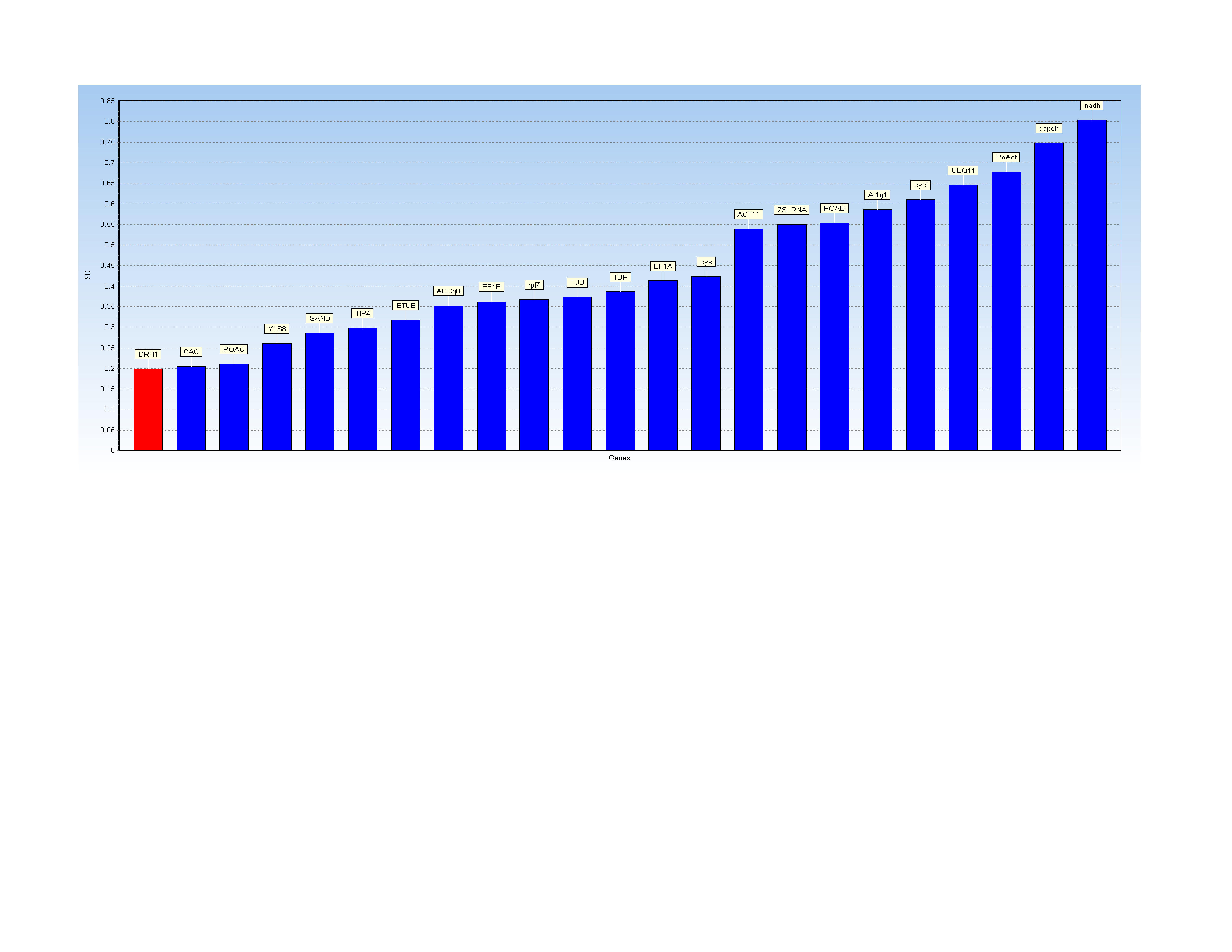

Supplement: Figure S1 — Standard deviations (SD) for stability ranking of reference genes calculated by NormFinder. One bar per gene represents the gene variability. The best reference gene is highlighted in red. (TIFF) [file pone.0068228.s001.tiff]

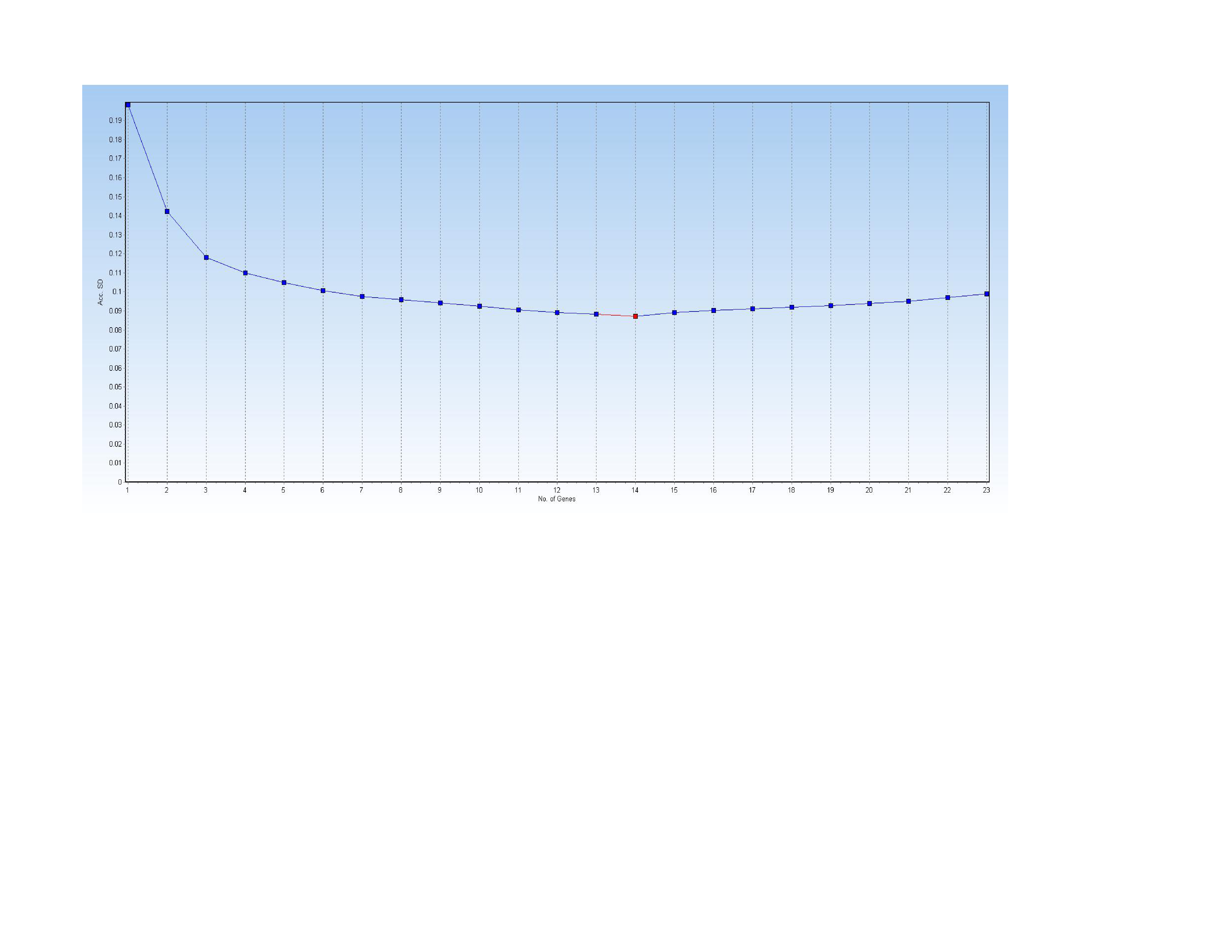

Supplement: Figure S2 — Determination of the optimal number of reference genes based on Acc. SD calculated by NormFinder. The Acc. SD is plotted against number of reference genes in a line plot where the optimal number of reference genes is indicated in red. (TIFF) [file pone.0068228.s002.tiff]
