# Supplementary material for: Evaluation of Reference Genes for RT-qPCR Expression Studies in Hop (Humulus lupulus L.) during Infection with Vascular Pathogen Verticillium albo-atrum
Source: PLoS One. 2013 Jul 12;8(7):e68228. doi: 10.1371/journal.pone.0068228 (PMC3709999; doi:10.1371/journal.pone.0068228)
Supplement: Table S1 — Amplicon sequences of 21 reference genes which were amplified in qPCR. (DOC) [file pone.0068228.s003.doc]

| ***Gene abbr.*** | ***Amplicon sequence*** | ***Amplicon size*** |
| --- | --- | --- |
| ***ACT11*** | GTGTCAGCCACACTGTTCCAATTTATGAGGGGTATGCCCTCCCACATGCTATCCTTCGACTTGACTTGGCAGGTCGTGAC | 80 bp |
| ***TUB*** | GAGACAAGTTCAGGCAAGCATGTGCCTAGAGCTATCTTTGTTGACTTGGAACCCACCGTTATCGACGAGGTTAGAACCGGCAAGTACCGC | 90 bp |
| ***UBQ11*** | GGTTCGGTCTCAGCTTTTGGGGTTGGCGTGTTTTGATGGGCTTATTAGTTGTTAAGCCTTTTGCAGAAAATAA | 73 bp |
| ***EF1B*** | GGGTATCAGGCTTCCAAGGATGATATCACTGTTCATGCTGCTATTTCAAAGGCTCCATCAGCAGACTACGTGAACGCGTCTCGTTGGTAC | 90 bp |
| ***ΒTUB*** | GAATGGATTCCGAACAATGTGAAGTCCACTGTTTGTGACATCCCCCCTACTGGGTTGAAGATGGCTTCGACTTTCATCGGCAACTCCACA | 90bp |
| ***EF1A*** | CCCCAGGACATCGTGACTTTATCAAGAACATGATTACTGGTACCTCTCAAGCCGACTGTGCTGTCCTCATTATTGACTCCACCACCGGAG | 90bp |
| ***POAB*** | GTGACCGAGGTGTCCTTGCTAGGGCTTCTGGGGACTACGCCGTCGTTATCAGTCATAACCCGGACAATGGAACCTCCAGAGTCAAGCTTCCATCTGGTGC | 100bp |
| ***POAC*** | AAGACGATCGCATAGCGAGAATTTCCTCTGCCATTCGAGTGATCCCGAACTTTCCTAAGCCAGGGATTTTGTTCCAAGACATCACTACGTTGCTTCTGGA | 100bp |
| ***POACT*** | TCGGTTGAGAAGAGCTACGAGTTGCCTGATGGACAGGTGATCACCATTGGCGCTGAACGTTTCCGTTGTCCAGAAGTCCTCTTCCAGCCATCCATGATCG | 100 bp |
| ***CYCL*** | CAGCCGGAAGGATCGTCATGGAGCTCTACGCCGATTGCGTTCCCCGCACCGCTGAGAACTTCCGTGCTCTGTGTACCGGTGAGAAGGGGATCGGAAAGAG | 100 bp |
| ***NADH*** | AA  GCTCCTTTCACTGCTTCTTACAGAAACTTCAAAGTCACGG  CCGCCGCTGCCGCCTCTACCGCCGCCAGGG GTTCTGGTAGGACATCTGAAGAGTTGGA | 100 bp |
| ***GAPDH*** | TGGAATGTCTTTCCGTGTTCCTACCGTCGATGTCTCAGTTGTTGACCTCACCGTCAGGCTTGCTAAGCCTGCATCCTACGAGGAGATCAAAAAAGCCATC | 100 bp |
| ***RPL7*** | TTCATTATTCGCATCCGTGGTATTAATGCCATGCACCCCAAGACAAGGACTATCTTGCAGCTCTTACGATTGAGACAGATATTCAATGGTGTGTTCCTTAA | 101 bp |
| ***CYS*** | CTGAGCAAGAGTTGGTGGATTGTGATACTTCTTACAACCAGGGCTGCAATGGTGGCCTCATGGACTATGCCTTCGAATTTATCATCAACAATGGCGGCAT | 100 bp |
| ***TBP*** | AACCAGACTCAACGGTCGTCTTAATCTAATCTTCTTTAGGACACGGCCGAATCATATCACTAACCTCAAGCTCCTCGCTTTCACGGTGAG TTGTCAATCG | 100 bp |
| ***TIP41*** | GGAGGTGGAGGTTGACGATAACGAGCTCAAGGCCGCTGGAGCCGAGCTTCTCACTGACGGACGTCGTGGCATCCGTATCCATGGTTGGGAGGTCGAGTCC | 100 bp |
| ***SAND*** | ACGAGCTGCTGTCGTTTGGGGGAGAACCATTTCGGAGATTGAGGTGGACGCGCCGGTCAGCCCCAGTAGTAGCGGGTATGCTGGTGAAAGAGGAAGTAGT | 100 bp |
| ***CAC*** | CTGGCCATGTGAGAATTTCCTTGATCTGCCCATTTTGGAGACCAACTTCTTGGTTTGAATTTTGCCAAAATTCTGCTGTTGTAGTGACAAAAATTGCGAG | 100 bp |
| ***ACCG8*** | GCAGCTTTTGCCTTTATACACTCAGATAGCTACTAAATTTGCAGAATTGCACGACACTTCCCAGAGGATGGCTGCAAAAGGAGTTGTTAAAGAAGTTCTGG AGTGGAC | 108 bp |
| ***YLS8*** | CGTACCTGCTTCCACATTTGCACTCTGGTTGGGCCGTAGATCAGGCCATCCTCGCCGAAGAAGAGCGTCTCGTCATCATCCGTTTTGGCCACGACTGGGA | 100 bp |
| ***AT1G1*** | AGCAAATCAATTGTACGAGCTTTGTGAAGCAGTGGGCCCTGAGCCAACCAGGACGGACTTGGTCCTGCATATGTGCGATTGCTTCCAGATAACGAGGCA | 100 bp |
