# Supplementary material for: Evaluation of Reference Genes for RT-qPCR Expression Studies in Hop (Humulus lupulus L.) during Infection with Vascular Pathogen Verticillium albo-atrum
Source: PLoS One. 2013 Jul 12;8(7):e68228. doi: 10.1371/journal.pone.0068228 (PMC3709999; doi:10.1371/journal.pone.0068228)
Supplement: Table S2 — The statistical analysis of data preformed with four factor ANOVA for fixed factors: cultivar (Celeia and Wye target), DPI (days post inoculation), infection (control and infected plants) and standardization (raw data, YLS8/DRH1 genes, 14 ref.genes, NADH gene). F and Pr values indicate that all interactions in four factor experiment are statistically significant. (DOC) [file pone.0068228.s004.doc]

|  | **Df** | **Sum Sq** | **Mean Sq** | **F value** | **Pr(>F)** |
| --- | --- | --- | --- | --- | --- |
| Cultivar | 1 | 6.76 | 6.76 | 636.849 | 0.0000 *** |
| Infection | 1 | 290.36 | 290.36 | 27338.22 | 0.0000 *** |
| Dpi (time) | 2 | 8.17 | 4.08 | 384.377 | 0.0000 *** |
| Normalization | 3 | 23.64 | 7.88 | 742.067 | 0.0000 *** |
| Cultivar:infect | 1 | 10.04 | 10.04 | 945.257 | 0.0000 *** |
| Cultivar:dpi | 2 | 16.66 | 8.33 | 784.142 | 0.0000 *** |
| Infect:dpi | 2 | 8.9 | 4.45 | 419.153 | 0.0000 *** |
| Cultivar:normalization | 3 | 0.42 | 0.14 | 13.095 | 0.0000 *** |
| Tret:normalization | 3 | 2.92 | 0.97 | 91.66 | 0.0000 *** |
| Dpi:normalization | 6 | 1.74 | 0.29 | 27.289 | 0.0000 *** |
| Cultivar:infect:dpi | 2 | 7.45 | 3.73 | 350.738 | 0.0000 *** |
| Cultivar:infect:normalization | 3 | 0.44 | 0.15 | 13.742 | 0.0000 *** |
| Cultivar:dpi:normalization | 6 | 1.49 | 0.25 | 23.31 | 0.0000 *** |
| Infect:dpi:normalization | 6 | 2.87 | 0.48 | 45.106 | 0.0000 *** |
| Cultivar:infect:dpi:normalization | 6 | 0.24 | 0.04 | 3.792 | 0.0020 ** |
| Residuals | 96 | 1.02 | 0.01 |  |  |
