# Supplementary material for: Evaluation of Reference Genes for RT-qPCR Expression Studies in Hop (Humulus lupulus L.) during Infection with Vascular Pathogen Verticillium albo-atrum
Source: PLoS One. 2013 Jul 12;8(7):e68228. doi: 10.1371/journal.pone.0068228 (PMC3709999; doi:10.1371/journal.pone.0068228)
Supplement: Table S3 — The average expression stability values of cultivars Celeia and Wye Target for infected (+) and control plants (−) at three different experimental time points (10, 20, 30 dpi). Expression values were normalized with three normalization factors obtained from two best reference genes (YLS8/DRH1), 14 best reference genes and least stable gene (NADH) and were compared to non-normalized (raw) values. se represent standard errors. Different letters indicate statistical significance based on Tukey’s multiple comparisons test of individual averages of expression levels. (DOC) [file pone.0068228.s005.doc]

| Normalization factor | Infection | DPI | **Celeia** | | | **Wye Target** | | |
| --- | --- | --- | --- | --- | --- | --- | --- | --- |
|  |  |  | Average | s_e_ | Statistical signif. | Average | s_e_ | Statistical signif. |
| raw_data | - | 10 | 6.8 | 0.1 | rst | 8.0 | 0.2 | qrs |
|  |  | 20 | 3.5 | 0.4 | v | 8.6 | 0.1 | pqr |
|  |  | 30 | 4.2 | 0.1 | uv | 0.9 | 0.1 | w |
|  | + | 10 | 116.0 | 2.2 | i | 17.7 | 0.2 | l |
|  |  | 20 | 110.5 | 10.5 | i | 144.7 | 8.8 | fghi |
|  |  | 30 | 118.0 | 6.0 | i | 40.8 | 0.9 | k |
| YLS8/DRH1 | - | 10 | 11.4 | 0.2 | nop | 19.4 | 0.4 | l |
|  |  | 20 | 5.7 | 0.7 | tu | 15.2 | 0.2 | lmn |
|  |  | 30 | 9.9 | 0.2 | opq | 4.1 | 0.3 | uv |
|  | + | 10 | 266.5 | 5.0 | c | 61.0 | 0.7 | j |
|  |  | 20 | 243.2 | 23.2 | cde | 176.3 | 10.7 | efgh |
|  |  | 30 | 118.0 | 6.0 | i | 44.6 | 0.9 | jk |
| 14_ref_genes | - | 10 | 9.1 | 0.2 | opqr | 16.4 | 0.3 | lm |
|  |  | 20 | 5.8 | 0.7 | stu | 12.4 | 0.2 | mno |
|  |  | 30 | 10.0 | 0.2 | opq | 4.6 | 0.3 | uv |
|  | + | 10 | 249.7 | 4.7 | cd | 53.6 | 0.6 | jk |
|  |  | 20 | 200.5 | 19.1 | cdef | 188.3 | 11.4 | defg |
|  |  | 30 | 135.3 | 6.8 | ghi | 58.7 | 1.2 | j |
| NADH | - | 10 | 16.4 | 0.3 | lm | 16.7 | 0.3 | lm |
|  |  | 20 | 3.5 | 0.4 | v | 16.0 | 0.2 | lmn |
|  |  | 30 | 18.2 | 0.3 | l | 5.4 | 0.4 | tu |
|  | + | 10 | 686.8 | 13.0 | a | 62.7 | 0.7 | j |
|  |  | 20 | 502.2 | 47.9 | ab | 466.0 | 28.2 | b |
|  |  | 30 | 445.8 | 22.6 | b | 130.9 | 2.7 | hi |
